# EXPLORING USE OF IMMERSIVE VIRTUAL REALITY IN OLDER ADULTS WITH COGNITIVE IMPAIRMENT

**DOI:** 10.1093/geroni/igae098.2899

**Published:** 2024-12-31

**Authors:** A’mie Preston, Kalpana Padala, Scott Mooney, Christopher Parkes, C Heath Gauss, Prasad Padala

**Affiliations:** Central Arkansas Veterans Healthcare System, North Little Rock, Arkansas, United States; Central Arkansas Veterans Healthcare System, North Little Rock, Arkansas, United States; Central Arkansas Veterans Healthcare System, North Little Rock, Arkansas, United States; Central Arkansas Veterans Healthcare System, North Little Rock, Arkansas, United States; University of Arkansas for Medical Sciences, Little Rock, Arkansas, United States; Central Arkansas Veterans Healthcare System, North Little Rock, Arkansas, United States

## Abstract

**Background:**

The innovative use of immersive virtual reality (iVR) has the potential to offer individualized, low-cost, accessible care to the older adult population. Virtual reality has gained tremendous interest for its potential uses with older adults who have cognitive impairment, though significant gaps still remain in the literature for this population. This research study aims to explore the feasibility, acceptability, and perception of technology for using iVR in an older adult, racially underrepresented, Veteran population with cognitive impairment.

**Methods:**

Assessments for participants and their caregivers explored mood, quality of life, cognitive functioning, neuropsychiatric symptoms (NPS) related to cognitive impairment, and perception of the iVR device. Veterans used a head-mounted iVR device during the study visit.

**Results:**

Participant demographics included race: Black (36.7%), White (63.3%). Participants reported enjoyment of iVR device [mean=111.7(± 14.05)]. Perception of iVR significantly changed after use (p=0.0001), with apparent improvement. Participants tolerated the experience well with an average score of 1.1 (± 1.34) depicted on the Simulation Sickness Questionnaire. No significant effect of the presence of NPS on the participants’ ability to immerse themselves in the iVR experience was found.

**Conclusion:**

Older adults can experience minimal symptoms during iVR use and may report high enjoyability. Reported NPS may not have a significant impact on the immersive experience of iVR. Perception of iVR can be positively improved following use of the device. Future research is needed in this area to identify use of iVR as a potential intervention for those with NPS associated with cognitive impairment.

